# Supplementary material for: The Small RNA Universe of Capitella teleta
Source: Front Mol Biosci. 2022 Feb 25;9:802814. doi: 10.3389/fmolb.2022.802814 (PMC8915122; doi:10.3389/fmolb.2022.802814)
Supplement: Supplementary file 1 [file DataSheet1.ZIP › Supplement/known/CAPTEscaffold_394_20720.pdf]

Provisional ID : CAPTEscaffold_394_20720
Score total : 2286.3
Score for star read(s) : 3.9
Score for read counts : 2280.1
Score for mfe : 1.3
Score for randfold : 1.6
Score for cons. seed : -0.6
Total read count : 4484
Mature read count : 4404
Loop read count : 0
Star read count : 80

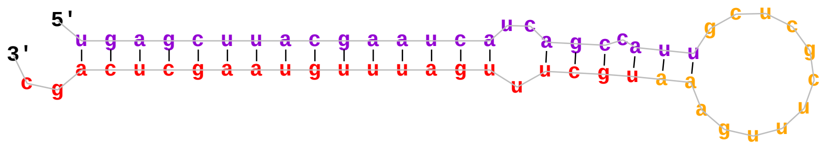

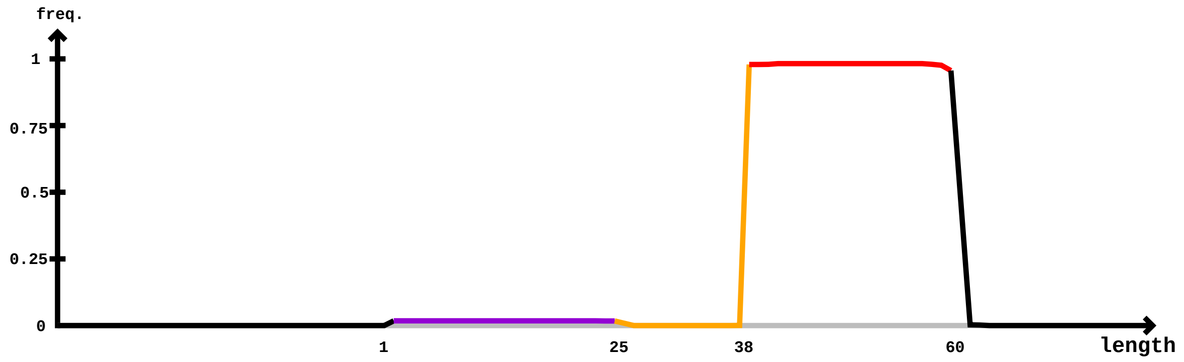

Star Mature

```
5'- caauucagcucagagaagacggaaccagugacuugagcuuacgaaucaucagccauugcucgcuuugaaaugcuuugauuuguaagcucagcaggcgugguuucaaaugcaa   -3'   obs
    caauucagcucagagaagacggaaccagugacuugagcuuacgaaucaucagccauugcucgcuuugaaaugcuuugauuuguaagcucagcaggcgugguuucaaaugcaa         exp
    caauucagcucagagaagacggaaccagugacuugagcuuacgaaucaucagccauugcucgcuuugaaaugcuuugauuuguaagcucagcaggcgugguuucaaaugcaa         known
    ....................(((((((((...((((((((((((((((..(((.(((...........)))))).))))))))))))))))...)).))))))).........    reads   mm   sample
    .................................ugagcuuacgaaucaucagcca.........................................................    3       0    seq
    .................................ugagcuuacAaaucaucagccauu.......................................................    1       1    seq
    .................................ugagcuuacgaaucaucagccauu.......................................................    74      0    seq
    .................................ugagcuuacgaaucauAagccauu.......................................................    1       1    seq
    .................................ugagcuuacgaaucaucagccauuU......................................................    1       1    seq
    .....................................................................Cugcuuugauuuguaagcucagc....................    2       1    seq
    ......................................................................ugcuuugauuuguaagcuc.......................    11      0    seq
    ......................................................................ugcuuugauuuguaagcuca......................    16      0    seq
    ......................................................................ugcuuugauuuguaagcucag.....................    90      0    seq
    ......................................................................ugcuuugauuuguaGgcucagc....................    83      1    seq
    ......................................................................ugcuuugauuuguaagcuGagc....................    1       1    seq
    ......................................................................ugcuuugaCuuguaagcucagc....................    1       1    seq
    ......................................................................ugcuuCgauuuguaagcucagc....................    1       1    seq
    ......................................................................ugcuuugUuuuguaagcucagc....................    1       1    seq
    ......................................................................ugcuuugGuuuguaagcucagc....................    1       1    seq
    ......................................................................ugcuuugauuuguaagUucagc....................    4       1    seq
    ......................................................................Cgcuuugauuuguaagcucagc....................    2       1    seq
    ......................................................................Ggcuuugauuuguaagcucagc....................    1       1    seq
    ......................................................................Agcuuugauuuguaagcucagc....................    10      1    seq
    ......................................................................Ngcuuugauuuguaagcucagc....................    4       1    seq
    ......................................................................uAcuuugauuuguaagcucagc....................    1       1    seq
    ......................................................................ugAuuugauuuguaagcucagc....................    1       1    seq
    ......................................................................ugcuuugaAuuguaagcucagc....................    1       1    seq
    ......................................................................ugcuuugauuuguaagcucagc....................    3971    0    seq
    ......................................................................ugcuuugauuuguaagcucagA....................    47      1    seq
    ......................................................................ugcuAugauuuguaagcucagc....................    3       1    seq
    ......................................................................ugcAuugauuuguaagcucagc....................    2       1    seq
    ......................................................................ugcuuugauuuguaagcuUagc....................    4       1    seq
    ......................................................................ugGuuugauuuguaagcucagc....................    2       1    seq
    ......................................................................ugcuuugauuuAuaagcucagc....................    1       1    seq
    ......................................................................ugcuuugauuuguaagcucagU....................    32      1    seq
    ......................................................................ugcuuugauuuguaagcAcagc....................    1       1    seq
```

```
                                Star                                 Mature
caauucagcucagagaagacggaaccagugacuugagcuuacgaaucaucagccauugcucgcuuugaaaugcuuugauuuguaagcucagcaggcgugguuucaaaugcaa
......................................................................ugcuuuAauuuguaagcucagc....................    5     1     seq
......................................................................ugcuuugauuugCaagcucagc....................    1     1     seq
......................................................................ugcuuugauAuguaagcucagc....................    1     1     seq
......................................................................ugcuuugauuuguaagAucagc....................    4     1     seq
......................................................................ugcuuugauuAguaagcucagc....................    2     1     seq
......................................................................ugcuuugauuuguaaAcucagc....................    1     1     seq
......................................................................ugcuuuUauuuguaagcucagc....................    2     1     seq
......................................................................ugcuuugauuuguaagcucagca...................    2     0     seq
......................................................................ugcuuugauuuguaagcucagcU...................   75     1     seq
......................................................................ugcuuugauuuguaagcucagcC...................    3     1     seq
........................................................................cuuugauuuguaagcucagc....................    1     0     seq
........................................................................cuuugauuuguaagcucagcag..................    1     0     seq
.........................................................................uuugauuuguaagcucagc....................    1     0     seq
.........................................................................uuugauuuguaagcucagcag..................    2     0     seq
.........................................................................uuugauuuguaagcucagcagA.................    5     1     seq
.........................................................................uuugauuuguaagcucagcagU.................    4     1     seq
```
